# Twin Studies of Atopic Dermatitis: Interpretations and Applications in the Filaggrin Era

**DOI:** 10.1155/2015/902359

**Published:** 2015-09-10

**Authors:** Camilla Elmose, Simon Francis Thomsen

**Affiliations:** ^1^Department of Dermatology, Bispebjerg Hospital, 2400 Copenhagen NV, Denmark; ^2^Center for Medical Research Methodology, Department of Biomedical Sciences, University of Copenhagen, 2200 Copenhagen N, Denmark

## Abstract

*Aim*. The aim of this study was to conduct a systematic review of population-based twin studies of (a) the concordance and heritability of AD and (b) the relationship between AD and asthma and, furthermore, to reinterpret findings from previous twin studies in the light of the emerging knowledge about filaggrin and its role in the atopic march and provide suggestions for future research in this area. *Methods*. We identified all twin studies (published after 1970) that have calculated the concordance rate and/or the heritability of AD, or the genetic and environmental correlations between AD and asthma. *Results*. Reported concordance rates for AD ranged, respectively. From 0.15 to 0.86 for MZ and from 0.05 to 0.41 for DZ twins, with an overall ratio of MZ : DZ twins of approximately three. The heritability of AD was estimated to be approximately 75%, and the association between AD and asthma was around 85% explained by genetic pleiotropy. *Conclusions*. Genetic factors account for most of the variability in AD susceptibility and for the association between AD and asthma. Controversy remains as to whether the atopic diseases are causally related or whether they are diverse clinical manifestations of a common, underlying (genetic) disease trait. Future twin studies may help solve this enigma.

## 1. Introduction

Atopic dermatitis (AD) is a common, inflammatory skin disease that primarily affects small children. 50% of cases appear within the first year of life, while 95% of cases have an onset before the age of five [1]. AD is a heterogeneous disease [2]; moderate and severe cases are usually easy to recognize, although differential diagnoses such as Netherton syndrome and cutaneous lymphoma should be considered, unlike mild and intermittent cases, which are less easily identifiable. During the last decades, the prevalence of AD has increased markedly, and in many Western countries, as much as one-fifth of all individuals are affected during their lifetime [3]. It is generally accepted that the rise in prevalence of AD, at least in part, can be explained by widespread environmental hygienic changes occurring in the last part of the 20th century. These changes are thought to have led to pronounced changes in the human microflora impacting pre- and perinatally on the risk of AD and other atopic diseases via complex interactions with the host immune system [4].

Pathophysiologically, two (complementary) hypotheses are commonly evoked to explain the inflammatory lesions of AD. The first is the* immunological hypothesis*, which argues that AD results from an imbalance of T cells [5]. In the acute phase of AD, the Th2 cytokines IL-3, IL-4, IL-5, and IL-13 dominate leading to increased levels of IgE, whereas the Th1 differentiation is correspondingly inhibited. In chronic AD, however, IFN-*γ*, IL-17, and IL-22 are important [3], suggesting that both Th1 and Th2 cells are contributors to the pathogenesis of AD. The second hypothesis is the so-called* leaky skin barrier hypothesis*, which is based on the observation that individuals with mutations in the filaggrin gene (*FLG*), which encodes the epidermal structural protein filaggrin, are at increased risk of developing AD [6]. Notably, individuals with* FLG* mutations experience a disrupted skin barrier, phenotypically characterized by dry, fissured skin, facilitating penetration of allergens, immunological dysfunction, and consequently an increased risk of developing eczema. Up to 50% of all patients with AD carry* FLG* mutations, making it the strongest known risk factor for AD [6].

Importantly, individuals with AD have an increased risk of developing hay fever and asthma [13, 17]. The basis of this association is imperfectly understood, and controversy remains as to whether the atopic diseases are causally related or whether they are diverse clinical manifestations of a common underlying (genetic) disease trait. The leading theory, however, follows from the leaky skin barrier hypothesis: among individuals with epidermal barrier defects, the skin acts as the site of primary sensitization with secondary reactivity in the airways [18].

Twin studies have been used to examine the relative importance of genetic and environmental factors for AD and also for the relationship between AD and related atopic diseases. Particularly, the concordance rate of AD is consistently higher for monozygotic (MZ) than for dizygotic (DZ) twins [13], indicating that genetic factors play a major role in the development of AD. Furthermore, twin studies have shown that genetic factors explain a sizable proportion of the cooccurrence of AD and the other atopic diseases, primarily asthma [15]. However, several of these studies were conducted before the discovery of filaggrin and its role in the atopic march, and consequently, this important information has been lacking in the interpretation of these findings.

Twin studies are a well-known and efficient method of estimating the relative genetic and environmental contribution to various diseases. Twin studies assume that, in principle, MZ twins are perfectly genetically matched, while DZ twins, like ordinary siblings, share half their segregating genes. Furthermore, MZ twins are assumed to share environmental risk factors to the same extent as DZ twins, and therefore, by comparing MZ with DZ twins, it is possible to estimate the relative impact of genetic and environmental factors on a given disease. Furthermore, twin studies allow estimation of the degree of overlap between genetic and environmental factors for related diseases.

Herein, we present a systematic review of (a) population-based twin studies of the concordance and heritability of AD and (b) twin studies of the relationship between AD and asthma. Furthermore, we reinterpret the findings from these previous twin studies in the light of the emerging knowledge about filaggrin and its role in the atopic march and provide suggestions for future studies in this area.

## 2. Methods

We performed searches in PubMed, Google Scholar, Scopus, and Embase combining the words “atopy,” “atopic dermatitis,” and “eczema,” with “heritability,” “concordance,” “correlation,” “twin,” and “twin study.” In a second search we combined the words “asthma” and “twin”/“twin study” with the words “eczema” and “atopic dermatitis.” We identified recent twin studies (published after 1970) that have calculated either the concordance rate for AD or the genetic and environmental correlations between AD and asthma. A total of 8 publications addressing the first question and a further 3 publications addressing the second question were identified. Each publication's reference list was scrutinized for missed cross-references.

### 2.1. Genetic Influence on Atopic Dermatitis

Several twin studies have estimated concordance rates for AD. Most of these have been based on questionnaire data, gathered through either parent-report, self-report, or both, depending on the age of the participants (Table 1).

In 1971, Edfors-Lubs [7] conducted a large population-based study comprising ~7000 twin pairs, aged 42–81 years, who were recruited through the Swedish Twin Registry. The study found concordance rates of 0.154 and 0.045 for MZ and DZ twins, respectively, indicating a significant influence of genetic factors on AD.

In a population-based study from 1986, Schultz Larsen et al. [8] examined a group of 592 like-sexed Danish twin pairs at school age, using questionnaire and clinical data. Participants were enrolled through the Danish Civil Registration System and were selected on the basis of affirmative responses to questions concerning AD as well as on the basis of clinical signs of AD. The probandwise concordance rates were 0.86 and 0.21, respectively, in MZ and DZ twins. In 1993, the same authors extended their findings by conducting a similar study [9] of 812 twin pairs, aged 8–22 years, using the same methodology: concordance rates were 0.72 and 0.23, in MZ and DZ twins.

In another population-based study of Swedish children (1480 twin pairs), 7–9 years of age, from 1997, Lichtenstein and Svartengren [10] found correlations in the liability to AD of 0.54 (MZ twins) and 0.35 (DZ twins) in boys, and 0.73 and 0.40 in girls, respectively, demonstrating a difference between sexes with a larger ratio of MZ to DZ concordance rates of eczema in girls. The study concluded, based on these differences in concordance rates, that, in boys, 75% of the individual susceptibility to AD was ascribable to additive genetic differences, whereas the remaining 25% was due to nonshared environmental factors. However, in girls, there was also an indication of an effect of shared environment (16% of the variation), which was, however, not statistically significant.

In a study from 2001, Strachan et al. [11] studied 873 female twin pairs, aged 18–72 years recruited through the St. Thomas UK Adult Twin Registry. The study found concordance rates of 0.51 and 0.47 in MZ twins below and above 50 years of age, respectively. For DZ twins, the same rates were 0.34 and 0.09. The St. Thomas Twin Registry comprises mainly female twins recruited through media campaigns.

In 2005 Nystad et al. [12] examined Norwegian twins in a population-based study of 3334 pairs recruited from The Medical Birth Registry using the International Studies of Asthma and Allergies in Childhood (ISAAC) questionnaires [3]. In MZ twin pairs, concordance rates of 0.43 and 0.44 were found in boys and girls, respectively, whereas for DZ twin pairs the corresponding rates were 0.06 and 0.10, thus demonstrating a greater MZ/DZ ratio in boys than in girls.

In 2007, Thomsen et al. [13] conducted a study of 11515 Danish Twin pairs, 12–41 years of age. Subjects were enrolled through the Danish Twin Registry. The study reported concordance rates of self-reported AD of 0.57 in MZ twins and 0.21 in DZ twins corresponding to a heritability of AD of around 80%.

Finally, a study by Jäderberg et al. from 2012 [14] examined a group of 9694 Danish twin pairs, 3–20 years of age recruited through the Danish Twin Registry, aiming to determine the impact of assisted reproduction on the risk of atopic diseases. The study found that AD was equally prevalent in twins born after assisted reproduction and spontaneous conception, respectively, with genetic effects accounting for 91% and 86% of AD in these two groups of twins. The difference in heritability was not statistically significant, demonstrating no mediating effect of assisted reproduction on AD development.

### 2.2. Association between Atopic Dermatitis and Asthma

Only a few studies have examined the association between AD and asthma in twins (Table 2). Particularly, in the previously mentioned study of Swedish twin children from 1997, Lichtenstein and Svartengren [10] found a phenotypic correlation between AD and asthma of 0.30. As much as 85% of this correlation in liability between the two diseases was ascribable to genetic factors, while the remaining 15% was due to nonshared environmental factors.

Also, among Danish adolescent and adult twins, Thomsen et al. [15] found a phenotypic correlation between AD and asthma of 0.40 and furthermore showed that 81% of the proportion of phenotypic overlap between AD and asthma was due to genetic pleiotropy, the remaining 19% being due to environmental factors. Additionally, atopic diseases were significantly associated within the same individual with an OR of developing asthma if one had eczema of almost five, and also the risk of developing any atopic disease was dramatically increased in a subject if the subject's twin brother or sister was affected, either by the same disease or by another. The effect was more pronounced in MZ twins compared with DZ twins, indicating that asthma and AD are genetically related [15].

In 2007, van Beijsterveldt and Boomsma [16] reported a correlation between AD and asthma of 0.55, and that pleiotropy explained 82% of this correlation, the remaining 18% being explained by environmental factors. The study population consisted of 8633 5-year-old twin pairs and was recruited through the Netherlands Twin Register, which comprises volunteer members, representing about 50% of all multiple births in the Netherlands.

### 2.3. Strengths and Limitations of the Reviewed Studies

Most of the identified studies rely solely on questionnaire responses. Only Schultz-Larsen et al. [8, 9] and Strachan et al. [11] have used clinical examination in order to diagnose subjects with eczema. Accordingly, there is a great possibility of recall bias in most of the previous twin studies. In contrast, Strachan et al. [11] argue that the combination of clinical examination, involvement of parents, and strictly defined diagnostic criteria makes it unlikely that recall bias should have affected the results. A particular strength of this UK study is that all clinical examinations were made by the same dermatologist, thus excluding the chance of interobserver bias. An obvious weakness of the same study is that the twin sample does not represent the general population, as the St. Thomas Twin Registry comprises twins mainly recruited through media campaigns. The authors however argue that major volunteer bias is unlikely, since recruitment did not inform participants of the specific hypotheses being tested.

An obvious strength of the Danish, Swedish, and Norwegian studies is that they were performed in countries that enable extraction of representative samples of the general population, as very large and structured twin registries are available in the Nordic countries [19]. In contrast, the Netherlands Twin Register relies on volunteer members and thus only represents around 50% of multiple births in the Netherlands. Therefore the population in the study by van Beijsterveldt and Boomsma [16] does not represent the general population as well as the ones in the Scandinavian studies.

All of the reviewed publications are based on twin samples, which might intrinsically bias the results, as it cannot be excluded that twins per se constitute a* special* population. According to van Beijsterveldt and Boomsma [16], several factors cause twins to deviate from the general population. In many instances, twin deliveries occur before 37 weeks of gestation [20], and therefore twins have an increased risk of lung damage, which in turn may increase the risk of developing asthma [21]. Indeed, in the Dutch study there was an indication that the frequency of asthma in the study group exceeded that of the general population [16]. Conversely, there is also evidence that the prevalence rate of asthma may be lower in twins than in singletons, since growing up with a cotwin increases the chance of exposure to infections in early life, which in turn protects against the development of allergic diseases [22].

## 3. Discussion

Since 1970, a total of eight publications have examined the heritability of AD using twin data. Older studies are considered too small and biased, in part because of lack of registration systems in most countries [7]. For MZ twins, concordance rates range between 0.15 and 0.86, while for DZ twins, the corresponding numbers range from 0.05 to 0.41. However, as the concordance depends on the prevalence of the disease, and since the studies reviewed herein represent a period in which AD has increased markedly in prevalence, the* ratio* between MZ and DZ concordances seems a more appropriate measure of the genetic influence on AD. Although the reviewed studies vary in terms of age group, period, and country of origin, they all show that MZ twins are more concordant for AD than are DZ twins with a ratio between these concordances of around three, demonstrating great importance of genetic factors in the development of AD. The overall heritability of AD estimated from previous studies is around 75%.

Of note, twin studies of the association between AD and asthma have concluded that genetic pleiotropy explains as much as 85% of this association [10, 15, 16]. These findings suggest that AD and asthma, to a large extent, are genetically related. However, all studies of the association between AD and asthma were conducted before, or very shortly after, the discovery of the role of* FLG* mutations in the progression of the atopic march. Particularly, in 2006, pioneering work by Palmer et al. [6] revealed that two different loss-of-function variants in* FLG*, carried by around 9% of the European population, are strong predisposing factors for AD. Furthermore, it was observed that* FLG* mutations are also associated with asthma, but only in the context of AD. With this work and later confirmed in several subsequent independent populations [18] an emerging paradigm for understanding the progression of the atopic march was founded. In 2009, a systematic review and meta-analysis by van den Oord and Sheikh showed that, among individuals with* FLG* mutations, the risk of AD was increased about two times in family studies and almost five times in case-control studies [23]. Also the risk of asthma was increased, but only in those with coexistent eczema. These findings provide strong supporting evidence that* FLG* defects constitute an important risk factor both for the development of eczema and for the later progression to other atopic diseases.

The atopic march is the sequential progression of AD to asthma and allergic rhinitis during the first years of life. According to the leaky skin barrier hypothesis it is assumed that epidermal defects in genetically predisposed individuals result in primary percutaneous sensitization, which later leads to secondary reactivity in the airways following allergen or irritant exposure [18]. Several studies have indicated a close relationship between the atopic diseases on the individual level [17, 24], and a systematic review by Wüthrich [25] showed a lifetime risk of allergic rhinitis and asthma in individuals with AD of 40–60%. Thus, a close association between atopic diseases has long been known and before the discovery of filaggrin it was reasonable to believe that this association was ascribable to a common genetic background for the atopic diseases. However, the discovery that* FLG* mutations predispose to both AD, and also to asthma in the context of AD, renders it possible that the high degree of genetic overlap between AD and asthma found in twin studies represents a* causal* relationship between AD and asthma mediated through a filaggrin-deficient skin barrier rather than because of true genetic pleiotropy. Still, this association might only exist for certain types of asthma, primarily classical atopic asthma with early onset, whereas adult-onset asthma or nonatopic asthma may result from different pathways.

### 3.1. Directions for Future Research

The chain of events that links AD to asthma is complex and involves a multitude of hereditary and developmental factors that exert their effect in the context of environmental exposures. Today, in the “postfilaggrin era,” we have indications to believe that the demonstrated link between the different atopic diseases is mediated through a congenital skin barrier deficiency caused by mutations in important structural proteins of the skin, particularly filaggrin. The twin studies reviewed herein, and most other previous studies of singleton populations, however, are retrospective, mainly based on questionnaire data. Therefore, in the future, it would be interesting to examine the progression of AD to asthma and hay fever in prospective twin studies. In such studies,* FLG* variants as well as clinical data on the onset, severity, morphology, and anatomical distribution of AD should be included. Also,* FLG* null alleles are associated primarily with early-onset AD [26] and with persistence of AD from childhood into adulthood [27], but not with late-onset AD [28] although it is associated with certain anatomical patterns of adult AD, particularly foot and hand eczema [29]. However, whether FLG variants are associated with the progression of asthma to AD deserves further study.

Given the perception that the link between AD and asthma is causal it might also be interesting to address the leaky barrier hypothesis further in the context of defects in other structural epidermal and epithelial (airway or gut) proteins, apart from filaggrin. Such studies investigating other factors involved in barrier function might help explain why less than half of all patients with AD develop asthma and, conversely, why many without AD and* FLG* defects still develop asthma. Interestingly, some studies have suggested specific epithelial barrier proteins to have implications for asthma development, even in the absence of AD. For example, Hackett et al. [30] found that, in the airway epithelia of patients with asthma, the membrane expression of caveolin-1 was significantly lower compared to that of subjects without asthma. Importantly, reduced caveolin-1 expression was accompanied by loss of junctional E-cadherin and b-catenin expression, disrupted epithelial barrier function, and increased levels of the proallergic cytokine thymic stromal lymphopoietin. Twin studies might prove valuable in the further substantiation of these findings. An opportunity could be to study twin pairs* discordant* for barrier protein gene mutations in order to look for differences in atopic disease risk between the twins. Also, MZ twin pairs* concordant* for gene mutations associated with epithelial barrier dysfunction would serve as an interesting sample for studying gene-environment interaction in atopic diseases if MZ pairs discordant for environmental exposures can be identified. Moreover, studying twins would enable us to estimate to what extent mutated barrier protein genes account for the variance in AD and asthma susceptibility, that is, the proportion of heritability explained by* FLG* and other mutated barrier protein alleles.

If the progression from AD to asthma, at least in part, is caused by deficient epidermal proteins, another area that might be interesting to address are preventive strategies for these diseases. In 2010, Simpson et al. [31] studied the effect of early skin barrier protection in 22 neonates, all at high risk of developing AD. All subjects were treated with emollient therapy from birth, and the results suggested a protective effect of this treatment, with only 15% of subjects developing AD in the study group, compared with 30–50% in previous studies [32]. Recently, similar findings from two randomised controlled studies emphasize the relevance of these measures [33, 34]. Future twin studies might focus on the impact of skin barrier protection in the primary prevention of both AD and asthma.

Another key area of future research where twin studies are expected to be particularly valuable is epigenetics [35, 36]. Epigenetics is the study of heritable changes of a disease that are not caused by changes in the DNA sequence. Examples include DNA methylation, posttranscriptional histone modification, and modification of noncoding RNAs. As MZ twins are genetically identical, and also because they share many environmental exposures, it is puzzling that in many instances only one twin develops AD and asthma while the other remains unaffected or affected to a lesser degree. In epigenetics, proteins that attach to silencer regions of DNA will cause these regions to express differently, thus leading to differences in disease expression between individuals. Accordingly, such epigenetic studies of, for example, MZ twins discordant for AD and asthma might provide valuable clues in this context, as these mechanisms may partly explain the relatively high degree of discordance between MZ twins for these diseases [36].

Previous twin studies have revealed that both genetic [37] and nongenetic [38] factors determine the diversity of the gut microbiome. Also, twin studies have indicated an impact of a reduced microbial diversity in the development of conditions such as Crohn's disease [39] and Hodgkin lymphoma [40]. In relation to AD and asthma future twin studies should explore whether or not these findings also pertain to these diseases, and, particularly, whether reduced microbial diversity is a consequence of disease, its treatment, or a particularly hygienic environment. If relevant in this context, it would be interesting to examine the possibilities of using this knowledge in the prevention and treatment of AD and asthma.

## 4. Conclusion

This systematic review of twin studies showed that genetic factors account for most of the variability in AD susceptibility and also for the association between AD and asthma. However, controversy remains as to whether the atopic diseases are causally related or whether they are diverse clinical manifestations of a common, underlying (genetic) disease trait. Future twin studies may help solve this enigma.

## Figures and Tables

**Table 1:** Population-based twin studies of the concordance and heritability of atopic dermatitis.

Study	Country	Age (years)	*N* (pairs)	MZ rate	DZ rate	MZ/DZ ratio	Heritability (%)	Diagnostic method
Edfors-Lubs (1971) [7]	Sweden	42–81	6996	0.154	0.045	3.42		Self-report
Schultz Larsen et al. (1986) [8]	Denmark	7–21	592	0.86	0.21	4.1		Clinical examination
Larsen (1993) [9]	Denmark	8–22	812	0.72	0.23	3.13		Clinical examination
Lichtenstein and Svartengren (1997) [10]	Sweden	7–9	1339					Parent-report
Boys				0.54	0.35	1.54	74	
Girls				0.73	0.40	1.83	71	
Strachan et al. (2001) [11]	UK	18–72	873					Clinical examination
<50				0.51	0.34	1.50		
>50				0.47	0.09	5.22		
Nystad et al. (2005) [12]	Norway	18–35	3334				69	Self-report
Boys				0.43	0.06	7.17		
Girls				0.44	0.10	4.40		
Thomsen et al. (2007) [13]	Denmark	12–41	11515	0.57	0.21	2.71		Self-report
Boys							82	
Girls							80	
Jäderberg et al. (2012) [14]	Denmark	3–20	9694					Self-report/parent-report
Assisted reproduction							86	
Spontaneous conception							78	

MZ, monozygotic; DZ, dizygotic.

**Table 2:** Population-based twin studies of the association between atopic dermatitis and asthma.

Study	Country	Age (years)	*N* (pairs)	Phenotypic correlation	Proportion due to genetic factors (%)	Diagnostic method
Lichtenstein and Svartengren (1997) [10]	Sweden	7–9	1339	0.30	85	Parent-report
Thomsen et al. (2006) [15]	Denmark	12–41	11231	0.40	81	Self-report
van Beijsterveldt and Boomsma (2007) [16]	Netherlands	5	8633	0.55	82	Parent-report

Phenotypic correlation is the correlation between the individual liabilities to atopic dermatitis and asthma.

Proportion due to genetic factors is the proportion of phenotypic correlation explained by genetic factors shared between atopic dermatitis and asthma.
